# ESCRT machinery components are required for Orthobunyavirus particle production in Golgi compartments

**DOI:** 10.1371/journal.ppat.1007047

**Published:** 2018-05-03

**Authors:** Natalia S. Barbosa, Leila R. Mendonça, Marcos V. S. Dias, Marjorie C. Pontelli, Elaine Z. M. da Silva, Miria F. Criado, Mara E. da Silva-Januário, Michael Schindler, Maria C. Jamur, Constance Oliver, Eurico Arruda, Luis L. P. daSilva

**Affiliations:** 1 Center for Virus Research, Ribeirão Preto Medical School, University of São Paulo, Ribeirão Preto, SP, Brazil; 2 Department of Cell and Molecular Biology, Ribeirão Preto Medical School, University of São Paulo, Ribeirão Preto, SP, Brazil; 3 Molecular Virology of Human Infectious Diseases, University Hospital Tübingen, Institute of Medical Virology and Epidemiology, Tübingen, Germany; Division of Clinical Research, UNITED STATES

## Abstract

*Peribunyaviridae* is a large family of RNA viruses with several members that cause mild to severe diseases in humans and livestock. Despite their importance in public heath very little is known about the host cell factors hijacked by these viruses to support assembly and cell egress. Here we show that assembly of Oropouche virus, a member of the genus *Orthobunyavirus* that causes a frequent arboviral infection in South America countries, involves budding of virus particles toward the lumen of Golgi cisternae. As viral replication progresses, these Golgi subcompartments become enlarged and physically separated from Golgi stacks, forming Oropouche viral factory (Vfs) units. At the ultrastructural level, these virally modified Golgi cisternae acquire an MVB appearance, and while they lack typical early and late endosome markers, they become enriched in endosomal complex required for transport (ESCRT) proteins that are involved in MVB biogenesis. Further microscopy and viral replication analysis showed that functional ESCRT machinery is required for efficient Vf morphogenesis and production of infectious OROV particles. Taken together, our results indicate that OROV attracts ESCRT machinery components to Golgi cisternae to mediate membrane remodeling events required for viral assembly and budding at these compartments. This represents an unprecedented mechanism of how viruses hijack host cell components for coordinated morphogenesis.

## Author summary

The *Peribunyaviridae* is a large order of RNA viruses that are globally distributed and may cause human diseases. In this study, we analyzed the assembly pathway of Oropouche virus (OROV), a peribunyavirus of the orthobunyavirus genus that is the etiologic agent of a frequent arthropod-transmitted viral disease in Latin American countries. OROV is neurotropic and causes a debilitating febrile illness, which can progress to meningitis and/or encephalitis in some patients. We show that assembly of OROV involves budding of virus particles toward the lumen of Golgi cisternae. During this process, endosomal sorting complex required for transport (ESCRT) components are hijacked to Golgi membranes to participate in the morphogenesis of viral factories and are required for efficient release of infectious OROV particles. Although many enveloped viruses have evolved to usurp elements of the ESCRT machinery for assembly and budding, the data provides unprecedented evidence that ESCRT proteins may be relocated to Golgi compartments during viral replication, and that this machinery is required for assembly/egress of an *Orthobunyavirus*.

## Introduction

The *Bunyavirales* is one of the largest orders of RNA viruses, containing nine virus families, including the *Peribunyaviridae* with five genera, one of which is the *Orthobunyavirus* with 48 species [1]. The genus *Orthobunyavirus* comprises zoonotic arboviruses that cause different diseases in humans, varying from febrile illness and encephalitis caused by Oropouche virus (OROV) and La Crosse virus, to hemorrhagic fever caused by Ngari virus [2]. OROV is the etiologic agent of Oropouche fever, a frequent arboviral infection in South America countries, especially in the Amazon region of Brazil, Peru and Venezuela and with documented cases also in Panama, Suriname and Trinidad [3–5].

Similar to other *Bunyavirales*, OROV is an enveloped, single-stranded RNA virus composed of a tripartite genome. The large (L) segment encodes an RNA-dependent RNA polymerase (RdRp) that catalyzes both transcription and replication. The medium (M) segment encodes a polyprotein that is co-translationally cleaved in the endoplasmic reticulum (ER) to form the envelope glycoproteins Gc and Gn, and a nonstructural protein NSm. Gc (~110 kDa) and Gn (~32 kDa) are type I integral membrane proteins that form an heterodimer in the ER which is transported to the Golgi complex [6]. The small segment (S) encodes the nucleocapsid N (25–30 kDa) protein, an abundantly produced viral protein that oligomerizes and encapsidates the virus genome, and the nonstructural protein NSs. The N protein also interacts with RdRp, Gn, and Gc, playing a major role in virus assembly [2]. During infection, the *Orthobunyavirus* Bunyamwera induces the formation of unique viral compartments, also known as viral factories (Vfs) around the Golgi complex, the site where viral glycoproteins interact with N protein for virus particle assembly [2, 7, 8]. Indeed, Bunyamwera virus infection causes major remodeling of Golgi membranes, leading to alterations in the organization of the Golgi stacks [7, 9]. However, the host cell machinery involved in these membrane-remodeling events is unknown.

For most enveloped viruses, assembly and budding are closely linked processes that require the invagination of membranes away from the cytosol, followed by membrane severing. Such events may occur at the plasma membrane (PM), or in an intracellular compartment that will later fuse with the PM for virion release [10]. Similar “reverse topology” membrane-remodeling activities take place during the maturation of early endosomes into multivesicular bodies (MVBs) and require the Endosomal Sorting Complex Required for Transport (ESCRT) machinery [11].

In endosomes, the ESCRT machinery is composed of four main components (ESCRT-0, I, II and III) that commonly act in a sequential order. ESCRT-0 promotes cargo recruitment, ESCRT-I/II also bind cargo and initiates membrane invagination, while ESCRT-III facilitates membrane fission [11–13]. Finally, Vps4, an ATPase member of the AAA family, disassembles and recycles ESCRT-III components and is essential for sustained ESCRT machinery functioning in various processes [14]. Another important player is Alix/AIP1, a multifunctional protein that may act in parallel to ESCRT-I/II exerting activities such as cargo selection [13], membrane deformation [15, 16], and binding/recruitment of ESCRT-III subunits [17–19]. Many enveloped viruses have evolved to usurp elements of the ESCRT machinery for assembly and budding [10].

In the present study, we demonstrate that ESCRT machinery elements are recruited to the Golgi complex in OROV infected cells and are required in the late steps of the viral replication cycle. We show that OROV infection induces the formation of prominent Vfs by modifying the Golgi cisternae, which acquires an MVB appearance. Although these viral-induced compartments lack typical early and late endosome markers, they are enriched in Vps4 and Alix. ESCRT activity is required for OROV morphogenesis since impairment of the ESCRT-III recycling by overexpression of an ATPase-defective mutant of Vps4 (Vps4E/Q) produces enlarged Vfs, where Vps4E/Q accumulates. Additionally, depletion of Tsg101 or Alix significantly compromises Vf biogenesis and infectious OROV particle production. To our knowledge, this is the first report showing the recruitment of ESCRT components to the Golgi complex and their involvement in the assembly/egress of an *Orthobunyavirus*.

## Results

### The one-step replication cycle of OROV

**Fig 1:**
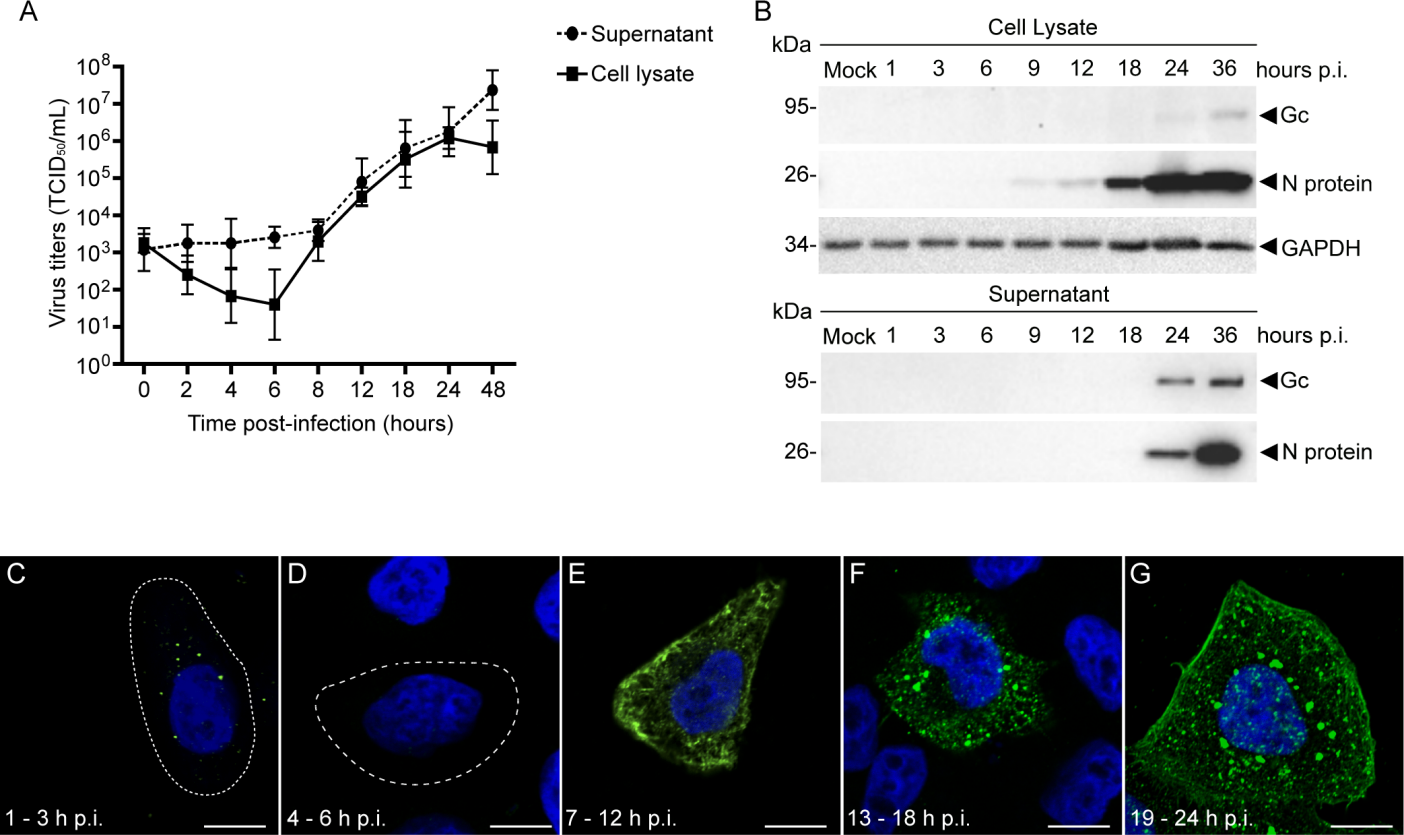
OROV replication cycle. HeLa cells infected with OROV were analyzed during the virus cycle. (A) Virus titers were determined by TCID_50_ assay in Vero cells. Data are the mean ± SD of three independent experiments and are displayed as TCID_50_/mL. (B) Detection of OROV proteins in cell lysate and supernatant at different times p.i. by immunoblotting. Primary antibodies to OROV proteins and GAPDH (loading control) were used and the molecular weight (in kDa) is indicated on the left. A representative immunoblot from three independent experiments is shown. (C-G) Subcellular distribution of OROV proteins (green), monitored by immunofluorescence and confocal microscopy analysis. The images shown are representative of the period p.i. indicated. Cell outlines are indicated by dashed lines. Nuclei were stained with DAPI (blue). Bars = 10 μm.

Initially, we monitored the kinetics of OROV replication cycle, in mammalian cells. To this end, HeLa cells were infected with OROV (MOI = 1) and the TCID_50_/mL from both cell lysate and supernatant samples were determined at different time points post-infection (p.i.). During the first 6 h p.i, the intracellular viral titers were continuously reduced and were barely detected, indicating virus eclipse (Fig 1A). This pattern was followed by a rapid increase in viral titers in cell lysates and culture supernatants, reaching peak levels in cell lysates at approximately 24 h p.i.

Viral cycle progression was also analyzed by determining the levels of OROV protein accumulation in cells and culture media by immunoblot. Using an anti-OROV polyclonal antibody the viral nucleocapsid (N) protein (MW ~25 kDa) was the most abundantly detected protein in cell extract and supernatant samples (Fig 1B). However, the glycoprotein Gc (MW ~124 kDa) was also detected at late times p.i. (Fig 1B). The immunoblot data are in agreement with the virus titration data by TCID_50_ assay, and help to define the times p.i. when viral proteins start to be produced and released.

Next, we assessed the subcellular distribution of OROV proteins in host cells during the replication cycle. At approximately 1–3 h p.i., viral proteins presented a puncta distribution dispersed throughout the cytoplasm (Fig 1C), likely representing endocytosis of viral particles as previously suggested [20]. Between 4–6 h p.i., virus staining could not be detected due to virus eclipse (Fig 1D). In contrast, after 7 h pi., viral proteins displayed a reticular pattern (Fig 1E), followed by accumulation in vesicle-like structures between 13–18 h p.i. (Fig 1F). These structures may represent the virus-induced cellular compartments that function as a scaffold for virus assembly, termed as viral factories (Vfs) [2, 21]. At later time points (19–24 h p.i.), these Vfs were still present, but an additional staining was also detected at the cell periphery (Fig 1G). Importantly, at 24 h p.i. almost all cells were positive for OROV without presenting a noticeable cytopathic effect. Taken together, these results indicate that the interval between 18 h and 24 h p.i. is a suitable period for studies on virus assembly site and budding.

### Endoplasmic reticulum membranes are recruited to the vicinity of Oropouche Vfs

Considering the reticular pattern displayed by OROV proteins at early times p.i. (7–12 h p.i., Fig 1E), we investigated the participation of ER membranes during the formation of Vfs. In non-infected cells, staining of calnexin-2, an ER-resident integral membrane protein responsible for folding glycosylated proteins [22, 23], showed a reticular pattern of distribution throughout the cytoplasm (S1A–S1D Fig). Although calnexin-2 labeling displayed poor overlap with OROV proteins (S1 Table), OROV infection changed the distribution of calnexin-2, as this ER-chaperone appeared to concentrate specifically at the vicinity of Vfs at 13–18 h p.i. (S1E–S1H Fig). This alteration in calnexin-2 distribution could be the result of changes in the trafficking of this transmembrane protein, or due to the direct recruitment of ER-membrane to Oropouche Vfs. To gain insights into this process, we investigate the behavior of a soluble ER-resident protein comprising the yellow fluorescent protein (YFP) fused to the ER-retention sequence, KDEL (SS-YFP-KDEL). Similar to calnexin-2, a clear enrichment of this lumenal ER-protein was seen in the vicinity of Oropouche Vfs (S1I–S1P Fig), strongly suggesting the active recruitment of ER-membrane to the OROV assembly sites.

### OROV assembles at enlarged Golgi cisternae

**Fig 2:**
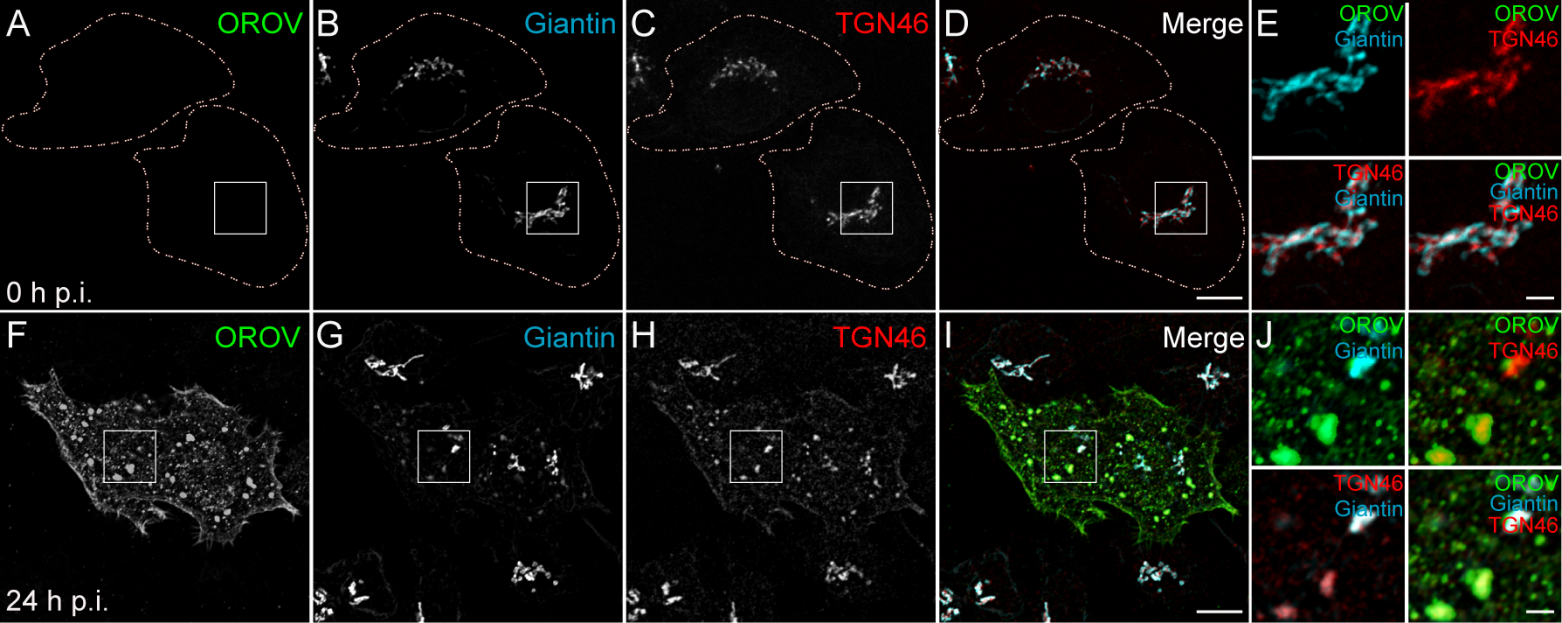
OROV recruits Golgi complex and TGN proteins to its assembly site. HeLa cells were infected with OROV and fixed after 0 h (A-D) or 24 h p.i. (F-I). Golgi complex and TGN proteins (Giantin and TGN46, respectively) were co-immunostained with OROV proteins and analyzed by confocal microscopy. Bars = 10 μm. (E and J) Insets representing the boxed areas of A-D and F-I, respectively. Bars = 2 μm.

It has been proposed that most orthobunyaviruses use the Golgi complex and/or the trans-Golgi network (TGN) as assembly sites in mammalian cells (Elliott 2014), which led us to analyze the role of these compartments during OROV assembly. To this end, we immunostained the *cis-*Golgi and the TGN using anti-giantin and anti-TGN46 antibodies, respectively, in OROV infected cells. Giantin is a coiled-coil protein that regulates Golgi architecture and function, by facilitating vesicle tethering and fusion processes in the cisternae [24]. TGN46 is thought to cycle between the TGN and the plasma membrane and at steady state is mostly detected on tubules and vesicles associated with the TGN [25]. In cells analyzed at 0 h p.i., TGN46 and giantin presented a juxtanuclear localization (Fig 2A–2E). In contrast, during OROV infection (24 h p.i.) these proteins were partially relocated to Vfs scattered throughout the cytosol, displaying colocalization with OROV staining at these structures (Fig 2F–2J and S1 Table). Taken together, these results indicate that, in addition to ER membranes, Golgi and TGN membranes contribute to the formation of Oropouche Vfs and that OROV infection induces the modification and scattering of Golgi elements.

**Fig 3:**
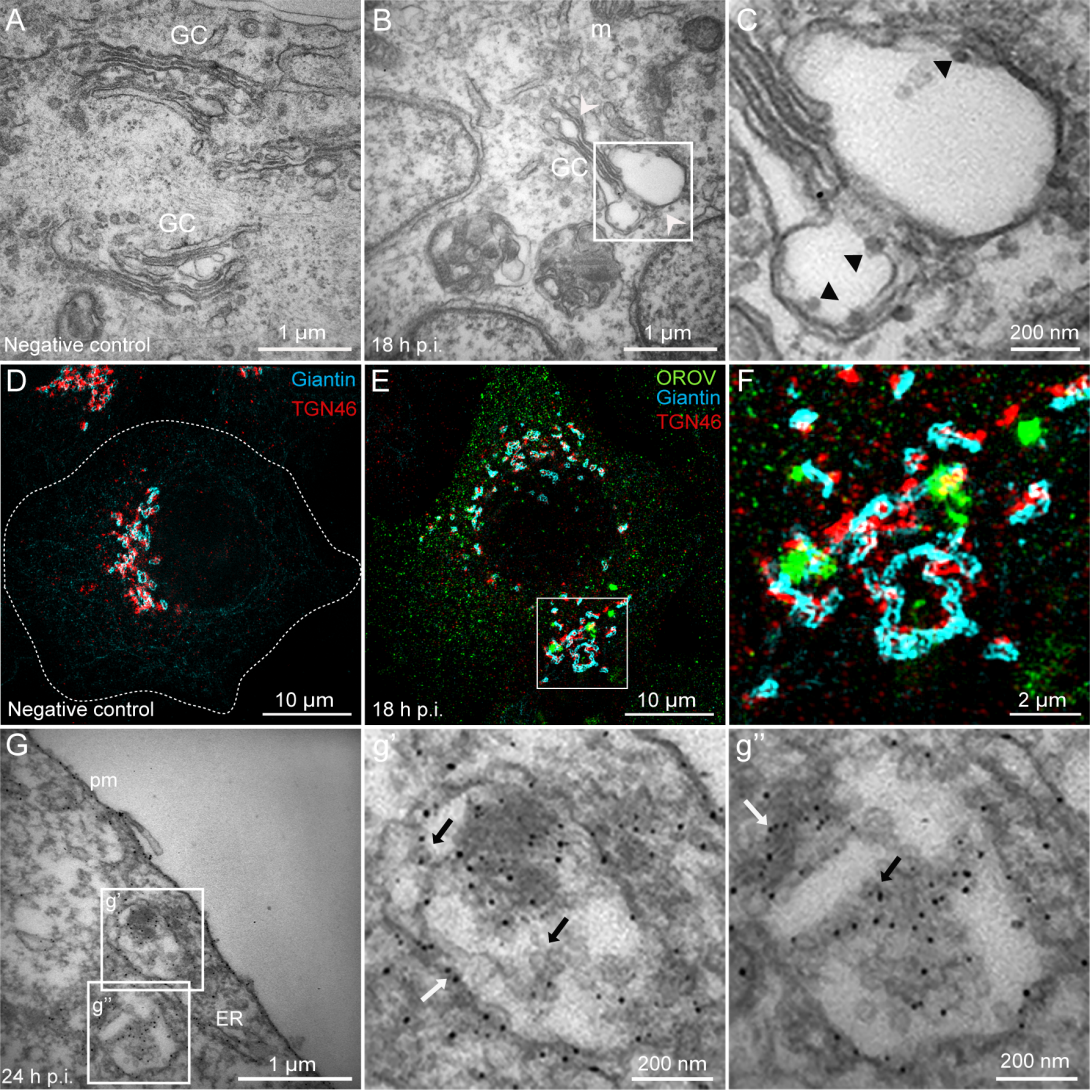
OROV viral factories derive from Golgi membranes. (A) Mock infected and (B) OROV infected HeLa cells at 18 h p.i.. White arrowheads show dilated Golgi cisternae. (C) Boxed area in (B) showing virus inside the Golgi cisternae (black arrowheads). 3D-SIM of mock infected (D) and OROV-infected (E) HeLa cells imaged at 18 h p.i. Cells were stained to OROV, TGN46 and giantin proteins, as described in Fig 2. The images represent projections of Z stacks (125 μm each) of cells after deconvolution. (F) Inset representing the boxed area of E. (G) Immunoelectron micrograph using a polyclonal anti-OROV antibody of an OROV infected cell at 24 h p.i. (g’-g”) Boxed areas in (G) showing virus staining on the vesicle membrane (white arrow) and viral particles inside the vesicle (black arrow). GC = Golgi complex; m = mitochondria; pm: plasma membrane; ER: Endoplasmic reticulum.

To obtain further insights into the morphological features of Oropouche Vfs, we analyzed HeLa cells infected with OROV at 18 h p.i. by transmission electron microscopy (TEM) and super-resolution microscopy. At this time of infection, prominent Vfs are starting to be detected (Fig 1E). In the non-infected cells, the Golgi complex was organized in thin parallel cisternae with numerous Golgi-associated vesicles at the proximity of the distal ends of the cisternae (Fig 3A). In infected cells, the Golgi cisternae appeared dilated and viral particles were observed within the lumen of these structures (Fig 3B and 3C). Moreover, the three-dimensional organization of Golgi and TGN cisternae in infected and non-infected cells was resolved by structured illumination microscopy (3D-SIM) and show that Vfs were commonly associated with Golgi and TGN membranes (Fig 3D–3F and S1 Movie). These EM and 3D-SIM results further support that the Golgi complex serves as platforms for the formation of viral compartments where OROV budding occurs. Oropouche Vfs were also analyzed by immuno-TEM using a polyclonal anti-OROV antibody at 24 h p.i. (Fig 3G, g’ and g”). This analysis revealed that Vfs constituted membrane-enclosed compartments with an endosomal-like morphology that resembles MVBs. OROV proteins were detected on the limiting membrane and inside of these compartments (Fig 3G, g’ and g”). Moreover, dilated ER cisternae were often detected in proximity to Vfs (Fig 3G).

### Expression of an ATPase-defective Vps4A mutant leads to the enlargement of Oropouche Vfs

The MVB appearance and the scattered distribution pattern of Vfs prompted us to investigate the presence of endosomal proteins in these structures. OROV staining presented a small colocalization with either CD63-GFP (~35%; S2A–S2H Fig and S1 Table) or Lamp1 (~10%; S2I–S2P Fig and S1 Table), proteins that are mostly localized to late endosomes/MVBs and lysosomes, respectively, and are widely used as markers for these compartments [26]. Similarly, OROV-staining rarely (~18%) overlapped with that of internalized transferrin (~18%), often used as a marker for recycling endosomes (S3A–S3H Fig and S1 Table) or of sorting nexin-2 (SNX2) (S3I–S3P Fig and S1 Table), a component of the retromer complex found in early endosomes [27]. Strikingly, we observed a higher degree (~64%) of colocalization between the OROV staining and that of HRS (S3Q–S3X Fig and S1 Table), a peripheral early endosomal protein that is a subunit of ESCRT-0 [28]. In fact, OROV Vfs were clearly enriched with HRS. Together, these results suggest that Vfs are not derived from early or late endosomes; rather, they indicate that the ESCRT-0 protein HRS is recruited to Golgi-derived Vfs during OROV biogenesis.

The enrichment of HRS in Oropouche Vfs prompted us to investigate the role of ESCRT machinery in OROV assembly and externalization. To avoid affecting virus internalization, HeLa cells were first infected with OROV (MOI = 3) and at 6 h p.i. cells were transfected with plasmids encoding GFP-tagged Vps4Awt or a dominant-negative Vps4A mutant (Vps4E/Q), in which the ATPase activity was compromised.

**Fig 4:**
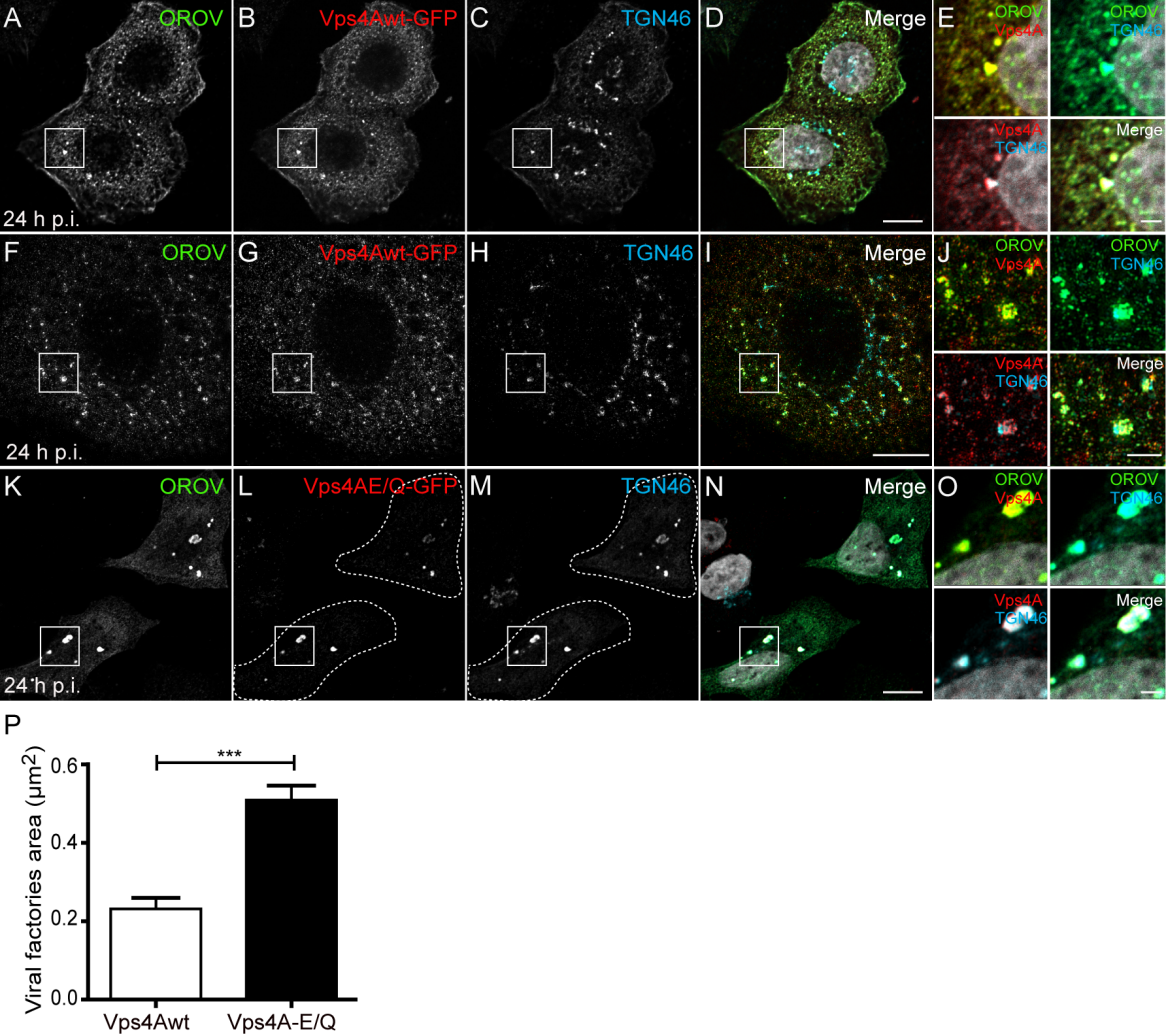
Vps4A is recruited to the TGN46 positive structures during OROV assembly. HeLa cells were infected with OROV and transfected with Vps4Awt-GFP (shown in red, A-J) or Vps4AE/Q-GFP (shown in red, K-O) plasmids. After 24 h p.i, cells were fixed, double stained with anti-OROV (shown in green to facilitate comparison with other Figures) and anti-TGN46 (Cyan) antibodies. (A-D) HeLa cells expressing Vps4wt-GFP were fixed and analyzed by conventional confocal microscopy. (F-I) 3D-SIM images of HeLa cells processed as (A-D). The image represents a projection of Z stacks (125 μm each) of cells after deconvolution. (K-N) HeLa VpsE/Q-GFP expressing cells were fixed after 24 h p.i and analyzed by conventional confocal microscopy. Bars = 10 μm. (E, J and O) Insets representing the boxed areas of A-D, F-I and K-N, respectively. Bars = 2 μm. (P) The areas of Vfs from least 15 cells for each condition from three independent experiments were determined using ImageJ software and are shown as mean ± SEM ***, P < 0.0005 (two-tailed paired *t-*test).

In non-infected control cells, Vps4wt-GFP displayed a cytosolic distribution (S4A–S4D Fig) as previously observed [29]. However, this pattern was altered by OROV infection, where the Vps4wt-GFP was redistributed mainly to Vfs (S4E–S4G Fig). By conventional confocal microscopy analyses, we noticed that Vps4wt-GFP appeared to be recruited to TGN46-positive structures that also contained OROV proteins (Fig 4A–4E). To analyze this phenotype with more detail, we used 3D-SIM and confirmed that Vps4wt accumulates in Oropouche Vfs associated with TGN46 (Fig 4F–4J and S2 Movie). Consistently, the ATPase inactive Vps4E/Q-GFP mutant strongly accumulated in TGN46/OROV-positive structures (Fig 4K–4O) and caused the enlargement of these structures (Fig 4P). Moreover, Vps4E/Q-GFP co-localization with TGN46 was clearly dependent of OROV infection (S4H–S4K Fig and S1 Table).

Next, we investigated the presence of specific viral molecules in the OROV-induced compartments containing Vps4. To this end, we expressed an OROV N-protein fused to mCherry (mCherry-N) in control and OROV infected HeLa cells. In control cells, mCherry-N showed a cytoplasmic distribution, localizing to discreet foci that may represent its association to membranes (S5A Fig). The pattern is different for Vps4-GFP, which is homogenously distributed in the cytoplasm and leaks out to the nucleoplasm, as previously shown (S5B Fig). In OROV infected cells, mCherry-N accumulates in larger structures to which Vps4-GFP is relocated and that are often found in the vicinity of TGN46-positive membranes (S5F–S5J Fig). It was previously shown that double stranded RNA (dsRNA), an intermediate of orthobunyavirus RNA replication, localizes to Golgi-derived viral tubes in Bunyawera infected cells [7]. S5K–S5O Fig shows that in non-infected cells, immunostaining for dsRNA is faint and is not spatially related to that of Vsp4-GFP. In contrast, a robust signal for dsRNA is detected in OROV infected cells, which partially overlaps with either Vps4-GFP alone (S5P, S5Q and S5S Fig arrows) or Vps4-GFP and TGN46 (S5P–S5T Fig).

Taken together, these results indicate that Vps4A is recruited to the TGN during OROV replication and its enzymatic activity is required for proper Oropouche Vfs biogenesis.

### ESCRT-I and Alix are required for Oropouche Vf morphogenesis and viral production

To further establish the involvement of the ESCRT machinery during OROV assembly, we used RNAi to reduce the expression of Tsg101 (a subunit of the ESCRT-I complex) or Alix (an ESCRT-accessory protein) in HeLa cells and monitored the production of infectious viral particles. Two distinct siRNA sequences targeting either Tsg101 (siTsg101#1 and siTsg101#2) or Alix (siTsg101#1 and siTsg101#2) were used in these experiments. A reduction of 94% (± 6%) and 95% (± 6.5%) in the expression of Tsg101 was achieved using siTsg101#1 and siTsg101#2, respectively. For Alix expression, a respective reduction of 80% (± 6%) and 81% (± 12%) was achieved using either siAlix#1 and siAlix#2.

**Fig 5:**
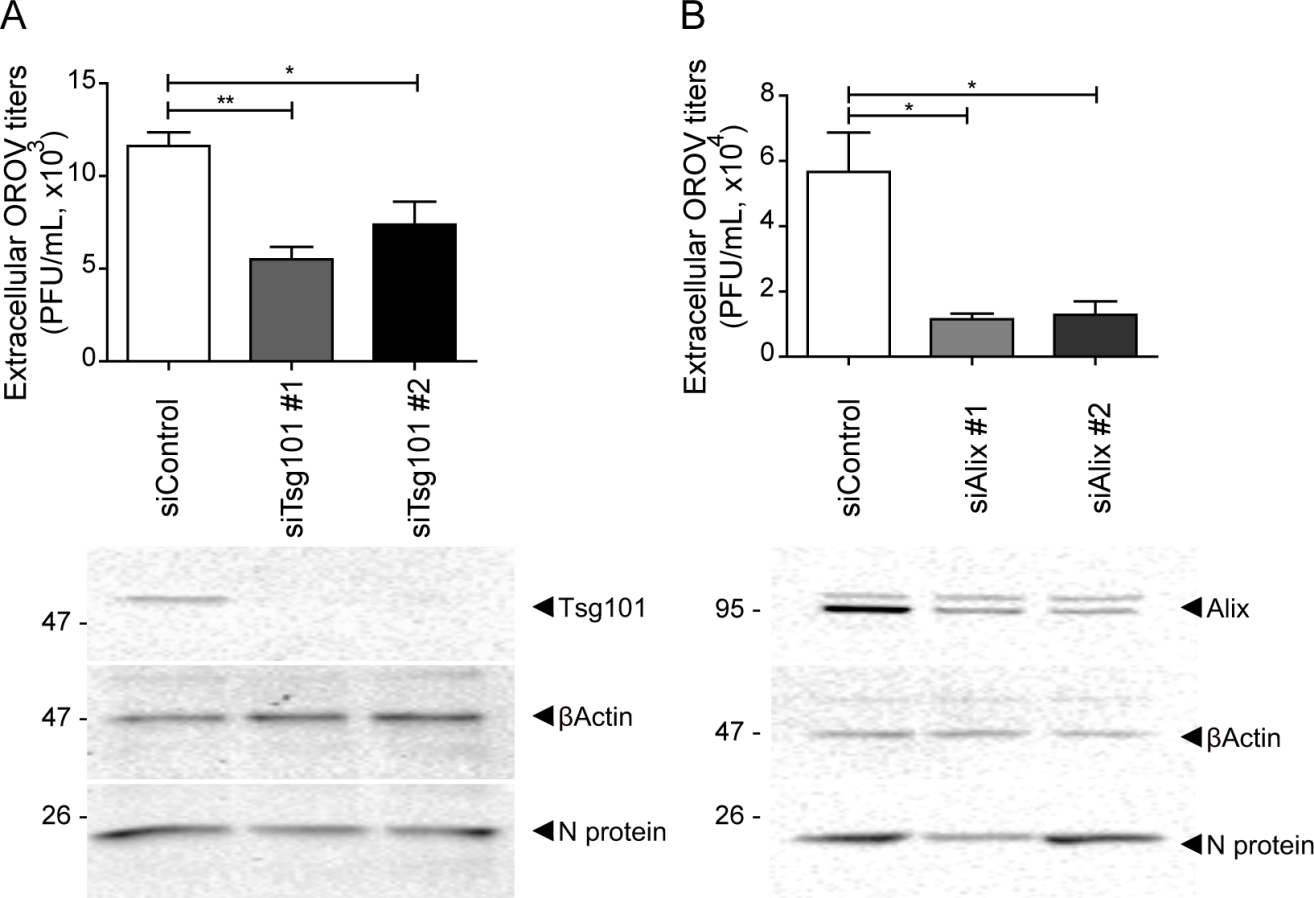
Knockdown of Tsg101 and Alix decrease the production of infectious OROV. HeLa cells were transfected with control, Tsg101 or Alix siRNAs and infected with OROV (MOI = 1). (A, B) After 18 h p.i, supernatant of infected cells were harvested and processed for PFU analysis (upper panels), and cell lysate were collected for western blot assay (lower panels). Supernatant samples were equalized according to the total amount of proteins in cell lysates. Two different siRNA sequences were used to deplete Tsg101 (A) or Alix (B) as indicated. Bars represent the relative mean ± SEM (n = 4 for panel A, and n = 3 for panel B). *, P < 0.05 **, P < 0.005 (one-way ANOVA followed by Bonferroni’s post-test).

Depletion of Tsg101, decreased the amount of infectious viral particles released in approximately 53% in cells treated with siTsg101#1, and ~37% in cells treated with siTsg101#2 (Fig 5A). Alix KD also decreased the total amount of infectious viral particles released by HeLa cells, a reduction of approximately 80% for siAlix#1 and 77% for siAlix#2 (Fig 5B).

**Fig 6:**
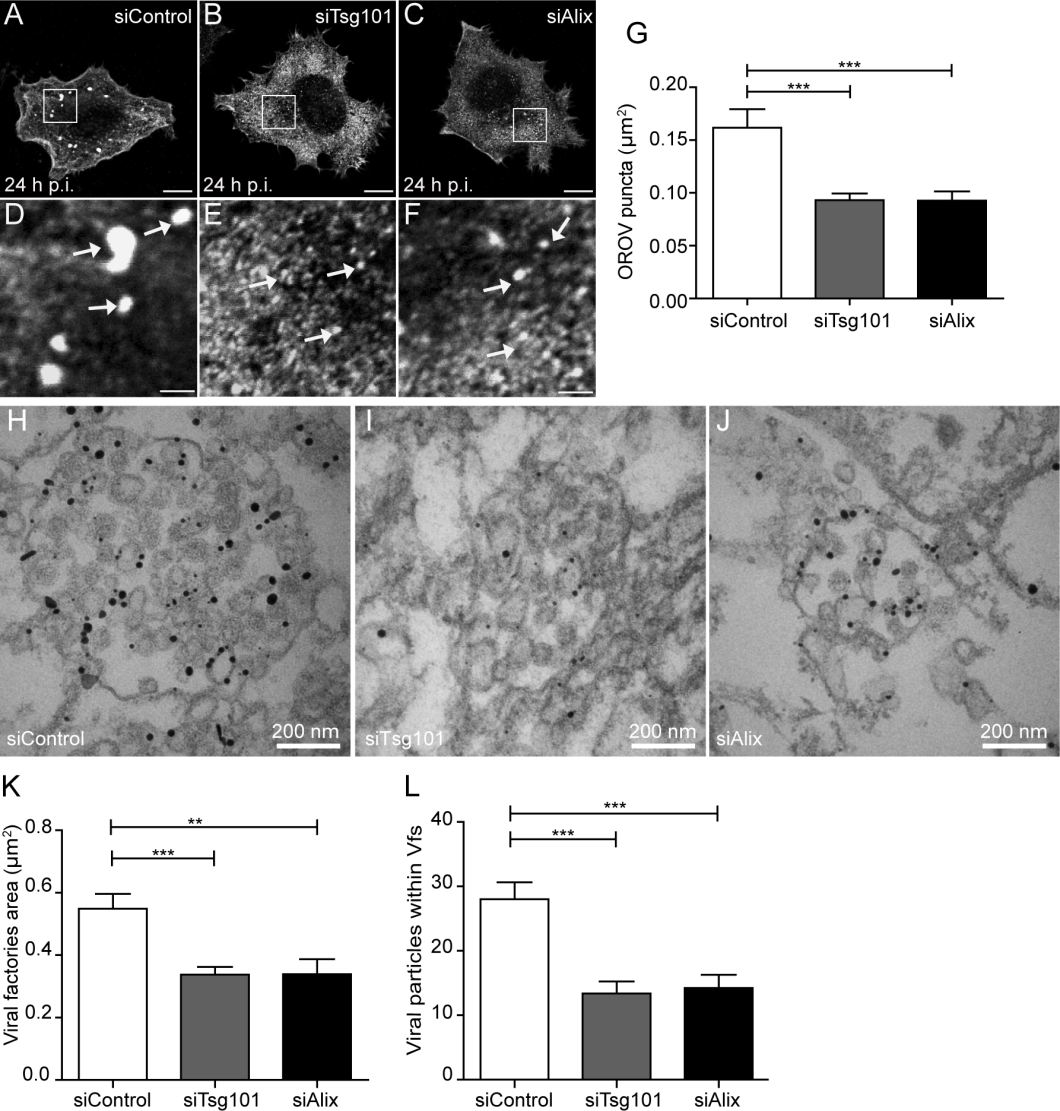
Knockdown of Tsg101 and Alix disrupt OROV assembly. HeLa cells were transfected with control, Tsg101#1 or Alix#1 siRNAs and infected with OROV (MOI = 1). (A-C) After 24 h p.i, cells were stained with anti-OROV antibody and analyzed by confocal microscopy. Bars = 10 μm. (D, E and F) Zoomed images of the boxed areas shown in A, B and C, respectively. White arrows indicate OROV Vfs. Bars = 2 μm. (G) The areas of Vfs from least 15 cells for each condition from three independent experiments were determined using ImageJ software (see Materials and Methods) and are shown as mean ± SEM. (H-J) After 12 p.i., cells were collected and processed for immuno-electron microscopy staining with anti-OROV antibody. (K, L) Area of the viral vesicles (K) and number of viral-like particles within viral vesicles (L) were obtained using ImageJ software (see Material and Methods). Bars represent the mean and ± SEM from a total of 13, 19 and 17 vesicles for siControl, siTsg101 and siAlix samples, respectively, in least 10 different cells for each condition. **, P < 0.005; ***, P < 0.0005. (two-tailed paired *t-*test).

Although knocking down Tsg101 or Alix did not block OROV entry process (S6 Fig), the depletion of either protein resulted in a significant reduction (~42% in both cases) in the area of the OROV vesicular structures observed at 24h p.i. (Fig 6A–6G). Finally, immuno-TEM analysis of Vfs labeled with anti-OROV antibody, revealed that depletion of either Tsg101 or Alix leads to smaller viral compartments that contain a significantly lower number of intraluminal viral-like particles, compared to cells transfected with control siRNA (Fig 6H–6L). Together these results indicate that the activity of Tsg101 and Alix are required for efficient OROV Vf morphogenesis and proper viral particle assembly.

**Fig 7:**
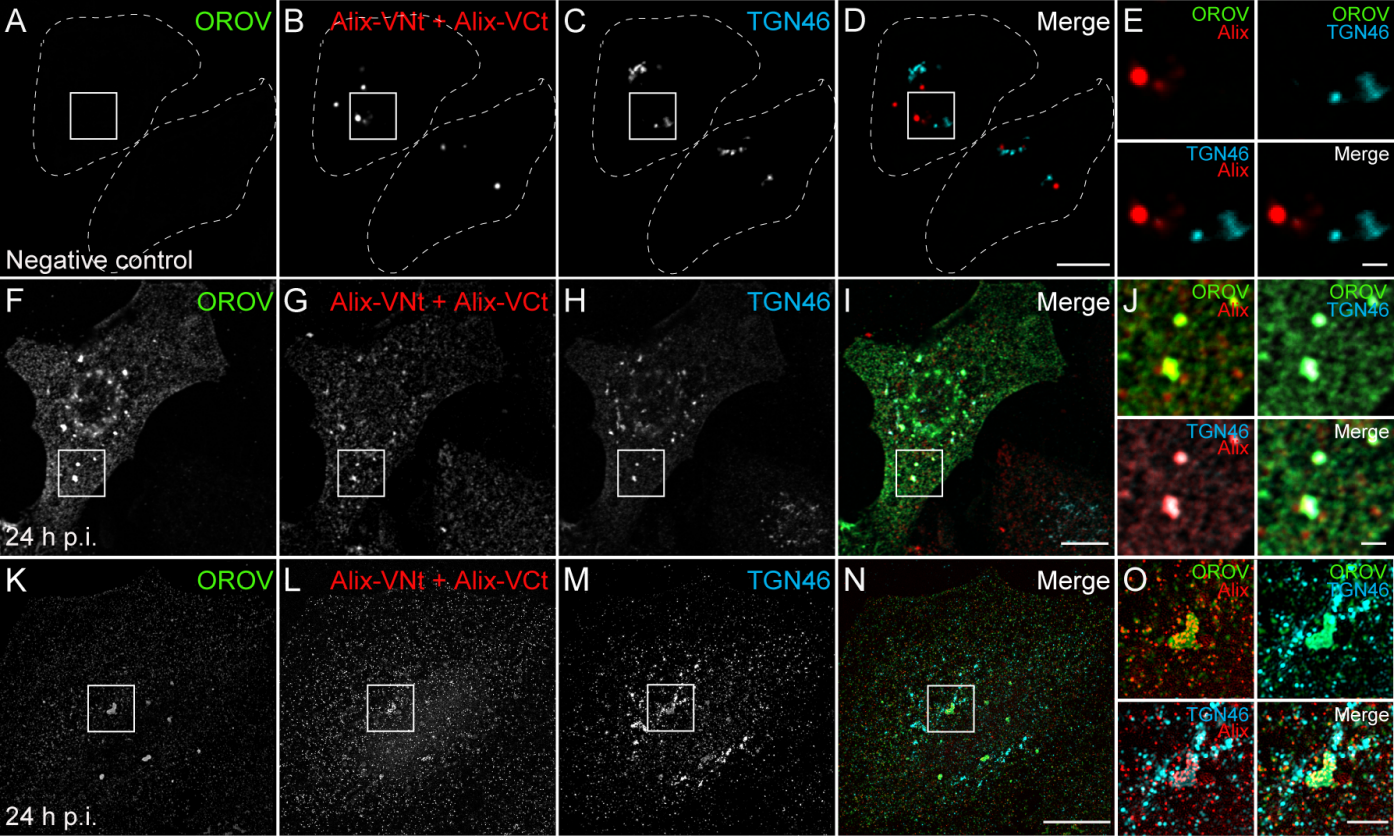
Alix is recruited to TGN during OROV assembly. Mock infected (A-D) or OROV infected (F-I) HeLa cells were transfected with VCt-Alix and VNt-Alix plasmids (red). Cells were immunostained to OROV proteins (green) and TGN46 (cyan) and analyzed by confocal microscopy. (K-N) 3D-SIM images of HeLa cells processed as (F-I). The image represents a projection of Z stacks (125 μm each) of cells after deconvolution. Bars = 10 μm. (E, J and O) Insets representing the boxed areas of A-D, F-I and K-N, respectively. Bars = 2 μm.

The experiments involving Alix depletion suggest that this protein may be recruited to Vfs for OROV replication. Alix dimerization was shown to be crucial for its association with endosomal membranes [30], and for mediating HIV budding [16]. To obtain further evidence for a role of Alix during OROV assembly, we used the bimolecular fluorescence complementation (BiFC) technique [31], in which the N- and C-terminal halves of a fluorescent protein are fused to Alix [30]. In non-infected cells, Alix-dimer/BiFC signal is mostly separated from TGN46 signal (Fig 7A–7E and S1 Table). In contrast, analysis of Alix-dimer formation in OROV infected cells by confocal microscopy revealed that Alix-dimers are strongly recruited to TGN46-positive structures at 24 h p.i. (Fig 7F–7J and S1 Table). Indeed, by 3D-SIM imaging, we confirmed that Alix-dimer signals clearly colocalize with TGN46 signals in OROV-positive structures (Fig 7K–7O and S3 Movie), and Alix-dimer/TGN46 colocalization is specifically induced by OROV infection (S1 Table). These results suggest that the ESCRT machinery components are recruited to Golgi/TGN membranes during OROV infection and provide additional evidence for a role of Alix in the biogenesis Oropouche Vfs. Taken together, our results show a novel critical requirement of the ESCRT machinery in post-entry events involved in the assembly and/or release of orthobunyaviruses.

## Discussion

The *Peribunyaviridae* is a large order of RNA viruses, which are widely distributed and may cause severe diseases in humans and cattle. However, prior to this work, there was no characterization of the cellular factors acting in assembly and budding of *Bunyavirales* in general. In this study, we analyzed the assembly pathway of the OROV, a neurotropic orthobunyavirus [32–34] that causes a frequent arthropod-transmitted viral disease in Latin American countries, with more than 500,000 cases confirmed in Brazil only [3–5]. We show that OROV induces the enlargement of Golgi cisternae, to where ESCRT machinery elements are recruited and support virion budding toward the lumen of this modified membrane-enclosed compartment.

For most *Bunyavirales*, viral particle formation is believed to start at the Golgi membranes, where the viral envelope glycoproteins (Gc and Gn), reaching this organelle from the ER, are retained [35]. At the Golgi membranes, the Gn cytosolic tail is thought to associate with viral ribonucleoproteins [36] comprised of the viral RNA segments, the RNA polymerase, and the nucleocapsid N-protein. In fact, studies with Bunyamwera virus, an orthobunyavirus considered a prototype of the genus *Orthobunyavirus*, indicated that the Golgi complex is the central organelle where viral particle assembly starts [9]. Further TEM and 3D reconstruction analyses of Bunyamwera virus infected baby hamster kidney (BHK-21) cells revealed the formation of large and complex Vfs at the juxtanuclear region [7]. It was determined, that these Vfs are composed of repetitive units constituted by one or more Golgi stacks, mitochondria, RER cisternae and virus-induced tubular membrane structures linking these organelles [7].

Here we provide evidence that OROV induces the formation of similar Vf structures in HeLa cells. However, OROV proteins do not appear to accumulate at the perinuclear region during the replication cycle. Rather, as infection progresses, OROV proteins concentrate in vesicular structures scattered throughout the cytoplasm (Fig 1). These structures are derived from enlarged Golgi cisternae (Fig 3) and are enriched in Golgi and TGN membrane marker proteins (Figs 2 and 3). Moreover, ER membrane domains appear to be actively recruited to the vicinity of these vesicular structures (S1 Fig). These results suggest that modified Golgi cisternae, in particular, the trans sub-compartments, are released from the Golgi stack and nucleate as physically separated Oropouche Vf units.

The results of this work also demonstrate a novel role for ESCRT machinery components in OROV Vf morphogenesis and virus production. The activity of ESCRTs in virus replication was initially identified, and is best characterized, for retroviruses [37–40]. Specifically, HIV-1 Gag can interact with both Alix and Tsg101, and use these proteins to recruit later ESCRT components to the plasma membrane for budding [10]. In fact, Tsg101/Alix co-depletion or impairment of Vps4 activity block HIV-1 budding [41, 42]. Currently, the requirement of ESCRTs for the efficient assembly and cell egress has been demonstrated for many other viruses [11, 40, 43–48]. However, a role of ESCRTs in *Bunyavirales* assembly and replication remained unknown.

In this study, we provide evidence that ESCRT machinery components are recruited to OROV replication sites in HeLa cells. First, we observed that endogenous HRS (an ESCRT-0 subunit), normally localized to early endosomes, appears to concentrate at viral replication sites (S2 Fig). Moreover, overexpressed Vps4wt, which is typically dispersed in the cytosol (S3 Fig), labels puncta structures that are also co-stained for OROV proteins and TGN46 in OROV infected cells (Fig 4 and S4 Fig). These virally induced Vps4wt-positive compartments were the sites of OROV N-protein accumulation and are often found in the vicinity of dsRNA, a component of the viral replication complex (S5 Fig). Although possible, the colocalization observed between either HRS or Vps4, and OROV proteins is unlike to represent mere leakage of OROV to endosomal compartments, because OROV staining display poor overlap with staining for markers for lysosomes (Lamp1), late endosomes/MVBs (CD63), recycling endosomes (transferrin) and for another early endosome protein (SNX2) (S2 and S3 Figs).

Further evidence for ESCRT recruitment to Vfs was provided using the ATPase-defective Vps4E/Q mutant. This mutant is still able to bind ESCRT-III and to associate with ESCRT-III positive membranes, but is unable to dissociate the complex [14]. Consistently with a role for the ESCRT machinery in OROV assembly, Vps4E/Q localization is strongly shifted to OROV replication sites where it colocalizes with TGN46 (Fig 4). Finally, using BiFC assays, we show that in OROV infected cells Alix-dimers assemble at TGN46-positive structures that are enriched in OROV proteins (Fig 7). Alix-dimer formation is crucial for Alix function as an ESCRT-accessory protein and generally takes place at endosomal membranes [30], but not at Golgi compartments (Fig 7). Besides implicating the ESCRT machinery in *Bunyavirales* assembly processes, these data are also relevant because they provide new evidence that ESCRT proteins may be relocated to Golgi compartments during viral replication. Interestingly, the ESCRT machinery has been recently reported to be recruited to the ER during the replication cycle of flavivirus [49], showing that the PM and endosomes are not the exclusive sites of ESCRT-activity during viral replication.

The function of ESCRT machinery elements is most likely required for OROV assembly and egress. Disturbing the ESCRT-III recycling via overexpression of an ATPase-defective Vps4 mutant leads to an enlargement of Vfs (Fig 4), indicating the importance of proper Vps4 enzymatic activity during OROV assembly. Moreover, depletion of either Alix or Tsg101 compromised Oropouche Vf formation, leading to smaller viral vesicles containing a lower number of intraluminal viral-like particles (Fig 6). Additionally, plaque forming units assays showed that the amount of infectious OROV particle production was reduced in cells depleted of either Tsg101 or Alix. Importantly, these detrimental effects observed for either Tsg101 or Alix depletion were not due a blockage in virus entry (S6 Fig). Therefore, our data strongly indicate that OROV requires ESCRT components for Vf biogenesis and the formation of infectious viral particles.

To conclude, this study reveals an additional function for the ESCRT machinery and contributes to clarify and amplify the current understanding of *Bunyavirales* replication cycle. Moreover, we identify a novel strategy of how viruses usurp and dysregulate the host cell machinery for efficient morphogenesis and egress.

## Materials and methods

### Cell culture and virus strain and propagation

Vero C1008 cells (American Type Culture Collection, Manassas, VA) and a previously described HeLa cell line (HeLa-I) that expresses ICAM-I on the cell surface [50], were maintained in Dulbecco’s modified Eagle medium (DMEM) supplemented with 10% fetal bovine serum (FBS, Thermo Scientific, Rockford, IL), L-glutamine and penicillin-streptomycin solution (Thermo Scientific). Cells were incubated at standard conditions (37°C with 5% CO_2_). OROV strain (BeAn19991) was propagated in Vero cells grown in DMEM supplemented with 2% FBS, L-glutamine and penicillin-streptomycin solution. Virus titers were measured by TCID_50_/mL assay as previously described [51].

### OROV one-step replication cycle

Subconfluent monolayers of HeLa-I cells were incubated with OROV stocks (MOI 1–3) for virus adsorption for 2 h on a rocker at 4°C. Monolayers were washed with ice-cold PBS and incubated with 0.5 ml of DMEM with 2% FBS in 5% CO_2_ at 37°C. At different times post infection (0, 1, 3, 2, 4, 6, 8, 12, 18, 24 and 48 hours) harvested cells and supernatants from triplicate monolayers were collected and used for quantification of viable virus by TCID50/mL and protein expression by immunoblotting.

### Cell transfections and RNA interference (RNAi)

The pEGFP-C2 Vps4A-wt and pEGFP-C2 Vps4A-E/Q (E223Q) plasmids were gifts from Philip Woodman (University of Manchester, United Kingdom). The pSS-YFP-KDEL (encoding the signal sequence of prolactin) plasmid was a gift from Jennifer Lippincott-Schwartz (NICHD, NIH, EUA). The pCD63-EGFP plasmid was kindly donated by Juan Bonifacino (NICHD, NIH, EUA). To generate the pmCherry-N plasmid, the OROV N-protein ORF was firstly amplified from pTVTOROVS (Acrani et al., 2015), a generous gift from Gustavo Acrani (Universidade Federal da Fronteira Sul, Brazil), and cloned into pmCherry-C2 (Takara Bio USA, Mountain View, CA). To prevent translation of NSs, which is encoded from a downstream AUG initiation codon on the same mRNA transcript as the N protein, we performed site-directed mutagenesis to introduce a T24C silent mutation that removes this alternative AUG in the pmCherry-N plasmid. For BiFC experiments, we used the pcDNA3.1/Zeo-VCt-Alix [52] and the pcDNA3.1/Zeo-VNt-Alix plasmid, generated as follows: the Alix open reading frame (ORF) was removed from pcDNA3.1/Zeo-VCt-Alix and used to replace the leucine zipper (LZ) sequence in the pcDNA3.1/Zeo-VNt-LZ [53]. This resulted in the VNt sequence (residues 1 to 158 of Venus protein) fused to a spacer sequence (GGGGSGGGGSSG), followed by the Alix sequence. HeLa cells transfections were performed using Lipofectamine 2000 (Invitrogen, Carlsbad, CA), as follows. For the pSS-YFP-KDEL and pCD63-EGFP plasmids, cells were first transfected and incubated for 12h for the proteins to reach a stead state and this was followed by infection with OROV (MOI = 3) for the times indicated in the legends. For the plasmids pEGFP-C2 Vps4A-wt and pEGFP-C2 Vps4A-E/Q cells were initially infected with OROV (MOI = 3) during 6 h to allow virus entry and viral protein expression, and then transfected and analyzed after 24 h p.i. The transfection of Alix-VNt and VCt-Alix plasmids were performed after 16 h p.i. (MOI = 3) and cells were harvested and analyzed after 24 h p.i.

siRNA were purchased from Sigma-Aldrich as nucleotide duplexes with 3′dTdT overhangs, designed to target human Tsg101 sequence #1 (5’-CCUCCAGUCUUCUCUCGUC-3’), Tsg101 sequence #2 (5’- CUCAAUGCCUUGAAACGAA-3’), Alix sequence #1 (5’-GCAGUAAUAUGUCUGCUCA-3’), and Alix sequence #2 (5’-GAACAAAUGCAGUGAUAUA-3’). The MISSION siRNA Universal Negative Control (SIC001, Sigma-Aldrich) was used in control experiments. HeLa cells were subjected to one round of siRNA (20 nM) transfection using Oligofectamine reagent (Invitrogen, Carlsbad, CA), according to the manufacturer’s instructions. After 24 h of siRNAs transfection, cells were infected with OROV (MOI = 1) and then collected for the analyses after 24 h p.i.

### Antibodies

The mouse anti-OROV antiserum (kindly donated by Dr. Luis Tadeu Moraes Figueiredo) was used for immunofluorescence, western blot, and immunoelectron microscopy assays. Rabbit polyclonal antibodies to Calnexin-2 (H-70; Santa Cruz, CA), Giantin (Covance, NJ) and Lamp-1 (Cell Signaling Technology, MA), sheep polyclonal antibody to TGN46 (AbD Serotec, Oxford), and J2 mouse monoclonal antibody (Mab) anti-double-stranded RNA (dsRNA; English & Scientific Consulting Kft, Hungary), were used for immunofluorescence assays. Polyclonal goat antibodies to Alix (N-20; Santa Cruz Biotechnology, CA), and mouse monoclonal antibodies to Tsg101 (BD Bioscience, CA), GAPDH (Sigma Aldrich, SG) and Actin (Ab-5; BD Bioscience, CA) were used for immunoblot experiments. Secondary antibodies conjugated to Alexa-fluorophores as indicated in the figure legends were purchased from Thermo Scientific (Rockford, IL). Horseradish peroxidase-conjugated donkey anti-mouse immunoglobulin G (IgG), donkey anti-rabbit IgG, and donkey anti-goat IgG were obtained from GE Healthcare.

### Plaque forming units (PFU) assay

Supernatant samples from knockdown assays were collected after 18 h p.i. and centrifuged by 3.000 g 10 min°C. Then, those samples were equalized according to the total amount of cellular proteins, measured by Bradford assay. Titration was performed on Vero E6 cells seeded at an 80–90% confluence in a 24-well plaque. Cells monolayer were infected with serial dilutions of virus samples for 1 hour at 37°C under slight agitation and then overlaid using 0.3% agarose in DMEM 2% FBS. 72 h post-infection, cells were fixed with 10% formaldehyde and stained using crystal violet to visualize plaques. Plaques were counted, and virus yield was calculated and expressed as PFU/ml.

### Immunofluorescence analyzes

Cells were seeded on 13-mm-diameter coverslips and fixed at the indicated times p.i. and processed for Immunofluorescence assay as previously described [54]. For dsRNA detection, using J2 antibody, samples were processed according to [55]. To detect recycling endosomes, we incubated serum starved OROV-infected HeLa cells with transferrin-Alexa 488 (life technologies) at 20 μg/mL in Opti-MEM for 30 min at 4°C. Then, cells were washed with ice-cold PBS to remove non-attached transferrin and incubated for an additional 1 h at 37°C. Cells were analyzed on a Zeiss confocal laser scanning microscope (LSM) 780 (Zeiss, Jena, Germany) or a Leica TCS SP5 laser scanning confocal microscope (Leica Microsystems, Wetzlar, Germany). Post-acquisition image processing and colocalization analysis were accomplished as previously described [52]. To determine the area of Vfs we used the analyze particles tools of the Fiji software [56], setting the lower and upper threshold levels of each image to 30,000 and 63,000, respectively. Alternatively, fixed cells images were acquired by a DeltaVision OMX SR system (GE Healthcare Life Sciences, Issaquah, WA, USA) by structured illumination microscopy (SIM). After deconvolution, projection of z-stacks (0,125 μm interval each), 3D sections or 3D reconstruction images were analyzed by Fiji software [56].

### SDS-PAGE and western blot analysis

Total cell lysates were prepared, equalized for total protein levels, and used for SDS-PAGE and Western blot, as described previously [52, 57]. To obtain virus lysates, cell culture supernatants were firstly clarified by centrifugation (2,000 x g at 4°C for 10 min) and then subjected to ultracentrifugation at 100,000 x g for 2 hours at 4°C through a 20% sucrose cushion. The pellets containing viruses were mixed with sample buffer [57], and proteins were resolved by SDS-PAGE followed by Western blot. Blots were probed with primary antibodies and HRP-conjugated secondary antibodies. Proteins in the blots were visualized using enhanced chemiluminescence solutions (GE Healthcare) and the ChemiDoc Imaging System equipped with the ImageLab software (Bio-Rad Laboratories, CA).

### Transmission electron microscopy

HeLa cells grown in 6-well plates were infected with OROV (MOI = 1) and collected at 18 h and 24 h p.i. Cells were then fixed in 2.5% glutaraldehyde in 0.1 M cacodylate buffer (pH 7.4) for 1 h and routinely processed for Electron Microscopy analysis as described further below. For pre-embedding immunoelectron microscopy, cells were processed as previously described [58]. Briefly, cells were fixed by microwave irradiation in 0.05% glutaraldehyde plus 4% formaldehyde in 0.1 M cacodylate buffer (pH 7.4) and subsequently immunolabeled with anti-OROV antibody and with goat anti-mouse IgG conjugated to nanogold (Nanoprobes). Cells were then fixed with 2.5% glutaraldehyde in cacodylate buffer for 1h before the nanogold was enhanced using GoldEnhance Electron Microscopy Plus (Nanoprobes) according to the manufacturer’s directions. In all TEM experiments cells were post fixed in 1% reduced OsO4 (Electron Microscopy Sciences) in 0.1 M cacodylate buffer (pH 7.4), rinsed in Milli-Q water, and dehydrated in a graded ethanol series. Cells were removed from the tissue culture plates with propylene oxide and embedded in EMBED 812 (Electron Microscopy Sciences). Thin sections were cut with a diamond knife, mounted on copper grids, and stained in Reynolds’s lead citrate and 0.5% aqueous uranyl acetate. Cells were imaged using a JEOL JEM-100CX II transmission electron microscope (JEOL USA, MA, EUA). To determine the area of Vfs we used the analyze particles tools of the Fiji software [56]. First we selected freehand tools to delimitate the viral vesicle, and then used measure tool to measure the vesicle area.

### Statistical analysis

All statistical data are demonstrated as mean ± SEM from at least three independent experiments (as indicated in each analyses). Statistical significance was calculated using unpaired t-test or One-way ANOVA followed by Bonferroni’s post-test, as indicated. P values are as displayed as follows: *P<0.05; **P<0.005; ***P<0.0005; ns, not significant. By convention, differences were considered statistically significant at P<0.05.

## Supporting information

S1 Fig OROV recruits ER membranes to its assembly sites. (A-H) HeLa cells were infected with OROV (MOI = 1) and analyzed at the indicated times post-infection. Cells were double stained with antibodies to OROV proteins (green) and Calnexin-2 (red). (I-P) HeLa cells were initially transfected with YFP-KDEL (red) plasmid and then infected with OROV (MOI = 1) for the indicated times post-infection (green). Cells were stained with antibody to OROV proteins (green) and analyzed by confocal microscopy. Cell outlines are indicated by dashed lines. Bars = 10 μm. (D, H, L and P) Insets representing the boxed areas of A-C, E-G, I-K and M-O respectively. Bars = 2 μm.
(TIF)

S2 Fig Late endosome and lysosome markers do not colocalize with OROV viral factories. (A-H) Cells were initially transfected with CD63-GFP (red) and then infected with OROV (MOI = 1) for the indicated times post-infection. Cells were stained with antibody to OROV proteins (green) and analyzed by immunofluorescence and confocal microscopy. (I-P) Control or infected cells (MOI = 1) were fixed at indicated times post-infection, and were double stained with antibodies to lysosome marker (Lamp-1) (red) and to OROV proteins (green) and analyzed by immunofluorescence and confocal microscopy. Cell outlines are indicated by dashed lines. Bars = 10 μm. (D, H, L and P) Insets representing the boxed areas of A-C, E-G, I-K and M-O respectively. Bars = 2 μm.
(TIF)

S3 Fig OROV recruits an ESCRT-0 component to its assembly site. (A—H) Control or infected cells (MOI = 1) were incubated with transferrin-Alexa488 (TRF, red) for one hour and then fixed at the indicated time p.i.. Cells were stained with antibody to OROV proteins (green) and analyzed by immunofluorescence and confocal microscopy. (I—X) Control or OROV infected cells (MOI = 1), were fixed at indicated times p.i., double stained with antibodies to early endosome markers (HRS and SNX2, shown in red) and to OROV proteins (green) and analyzed by immunofluorescence and confocal microscopy. Cell outlines are indicated by dashed lines. Bars = 10 μm. (D, H, L, P, T and X) Insets representing the boxed areas of A-C, E-G, I-K, M-O, Q-S and U-W respectively. Bars = 2 μm.
(TIF)

S4 Fig Vps4Awt and Vps4AE/Q do not overlap with TGN46 in the absence of OROV. (A-D) Control HeLa cells expressing Vps4Awt-GFP (shown in red) were immunostained with an anti-TGN46 antibody (cyan). (E-G) HeLa cells were infected with OROV (MOI = 3) and then transfected with Vps4Awt-GFP plasmid. After 24 h of infection, cells were fixed, double stained with anti-OROV (shown in green to facilitate comparison with other Figures) and anti-TGN46 (Cyan) antibodies. (H-K) Control HeLa cells expressing Vps4E/Q-GFP (shown in red) were immunostained with an anti-TGN46 antibody (cyan). Cells were analyzed by immunofluorescence and confocal microscopy. Cell outlines are indicated by dashed lines. Bars = 10 μm. (D and K) Insets representing the boxed areas of A-C and H-J respectively. Bars = 2 μm.
(TIF)

S5 Fig Vps4wt colocalizes with OROV nucleoprotein and dsRNA in OROV infected cells. (A-E) Control HeLa cells expressing Vps4Awt-GFP and mCherry-N of OROV were immunostained with an anti-TGN46 antibody (cyan). (F-J) HeLa cells were infected with OROV (MOI = 3) and then transfected with referred plasmids. After 24 h p.i, cells were fixed and stained with anti-TGN46 (cyan) antibody. (K-O) Control HeLa cells expressing Vps4wt-GFP were immunostained with a J2 anti-dsRNA (red) and an anti-TGN46 (cyan) antibodies. (P-T) HeLa cells were infected with OROV (MOI = 3) and then transfected with Vps4Awt-GFP plasmid. After 18 h p.i, cells were fixed and costained with J2 anti-dsRNA (red) and anti-TGN46 (cyan) antibodies. Cells were analyzed by immunofluorescence and confocal microscopy. Cell outlines are indicated by dashed lines. Bars = 10 μm. (E, J, O and T) Insets representing the boxed areas of A-D, F-I, K-N and P-S respectively. Bars = 2 μm.
(TIF)

S6 Fig Knockdown of ESCRTs components do not alter OROV entry. HeLa cells transfected with either control siRNA (A) or siRNA to Tsg101#1 (B) or Alix#1 (C) were infected with OROV (MOI = 3) for 7 h. Cells were immunostained with antibody to OROV proteins (green) and analyzed by conventional immunofluorescence microscope. Nuclei were stained with DAPI (blue). Bars = 100 μm. (D) The amount of infected cells (A–C) was calculated from the percentage of total cell count. ns–non significant (one-way ANOVA followed by Bonferroni post-test).
(TIF)

S1 Table Quantitative analysis of colocalization between OROV proteins and dsRNA, ER, TGN or endosomal proteins. (DOCX)

S1 Movie 3D reconstruction of the magnified area shown in Fig 3E. 3D reconstruction was performed with Fiji software. The virtual Z section is 125 μm. Green, OROV; Cyan, Giantin; Red, TGN46.
(MP4)

S2 Movie 3D section of the magnified area shown in Fig 4I. 3D sectioning was performed with Fiji software. The virtual Z section is 125 μm. Green, OROV; Cyan, TGN46; Red, Vps4wt-GFP.
(MP4)

S3 Movie 3D section of the magnified area shown in Fig 7N. 3D sectioning was performed with Fiji software. The virtual Z section is 125 μm. Green, OROV; Cyan, TGN46; Red, VNt-Alix + VCt-Alix.
(MP4)
