# Supplementary figures and images for: ESCRT machinery components are required for Orthobunyavirus particle production in Golgi compartments
Source: PLoS Pathog. 2018 May 3;14(5):e1007047. doi: 10.1371/journal.ppat.1007047 (PMC5953487; doi:10.1371/journal.ppat.1007047)

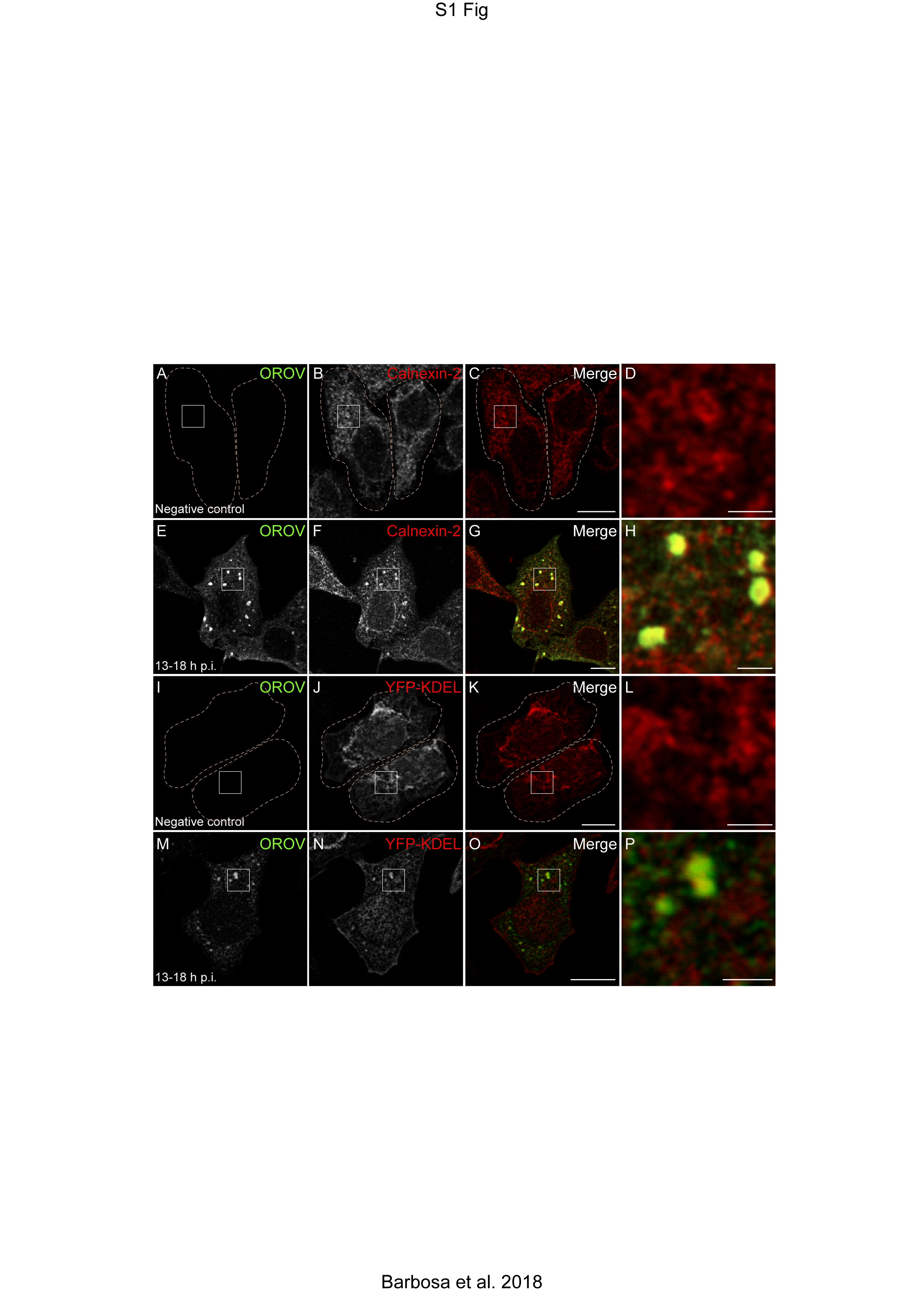

Supplement: S1 Fig — (A-H) HeLa cells were infected with OROV (MOI = 1) and analyzed at the indicated times post-infection. Cells were double stained with antibodies to OROV proteins (green) and Calnexin-2 (red). (I-P) HeLa cells were initially transfected with YFP-KDEL (red) plasmid and then infected with OROV (MOI = 1) for the indicated times post-infection (green). Cells were stained with antibody to OROV proteins (green) and analyzed by confocal microscopy. Cell outlines are indicated by dashed lines. Bars = 10 μm. (D, H, L and P) Insets representing the boxed areas of A-C, E-G, I-K and M-O respectively. Bars = 2 μm. (TIF) [file ppat.1007047.s001.tif]

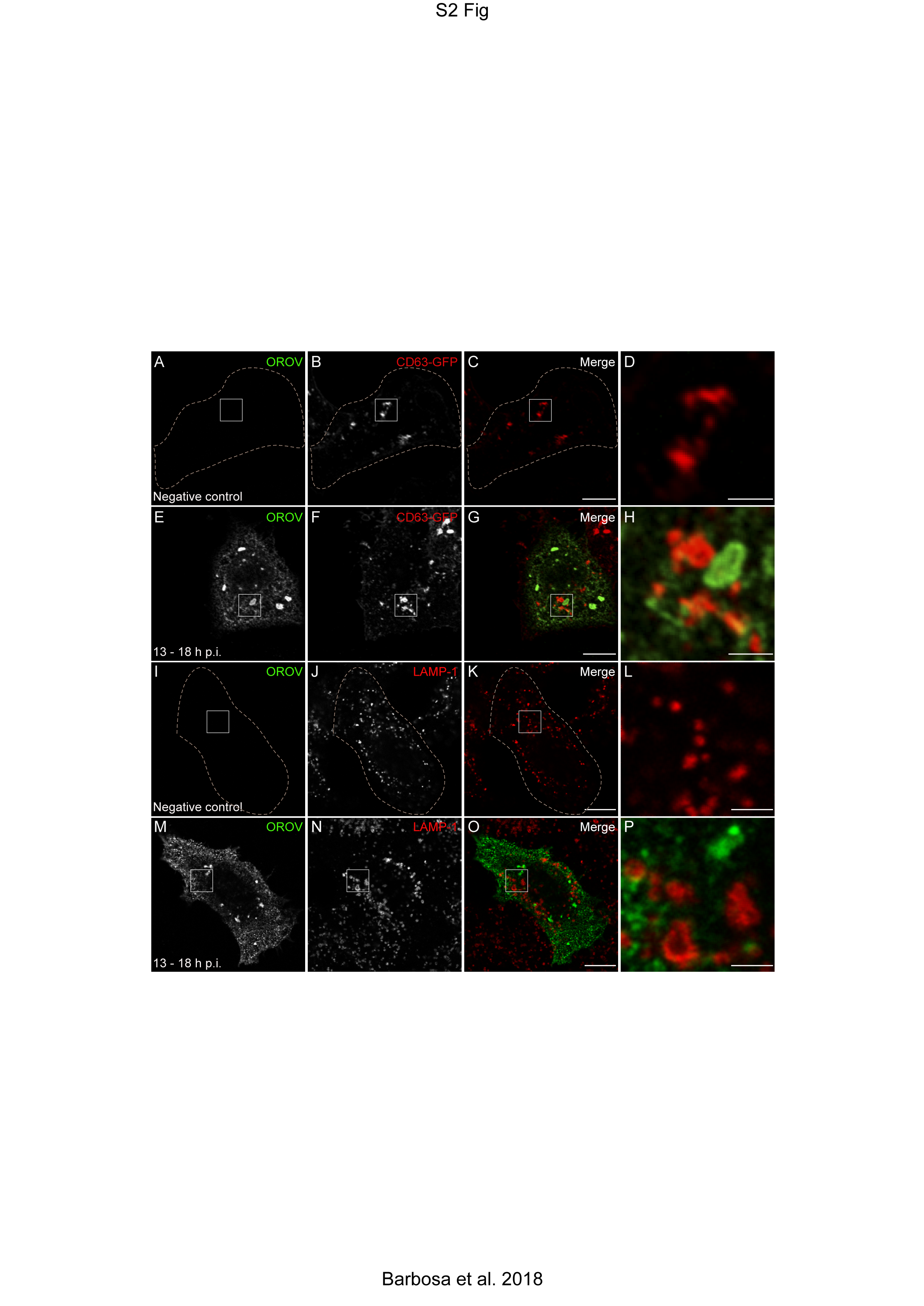

Supplement: S2 Fig — (A-H) Cells were initially transfected with CD63-GFP (red) and then infected with OROV (MOI = 1) for the indicated times post-infection. Cells were stained with antibody to OROV proteins (green) and analyzed by immunofluorescence and confocal microscopy. (I-P) Control or infected cells (MOI = 1) were fixed at indicated times post-infection, and were double stained with antibodies to lysosome marker (Lamp-1) (red) and to OROV proteins (green) and analyzed by immunofluorescence and confocal microscopy. Cell outlines are indicated by dashed lines. Bars = 10 μm. (D, H, L and P) Insets representing the boxed areas of A-C, E-G, I-K and M-O respectively. Bars = 2 μm. (TIF) [file ppat.1007047.s002.tif]

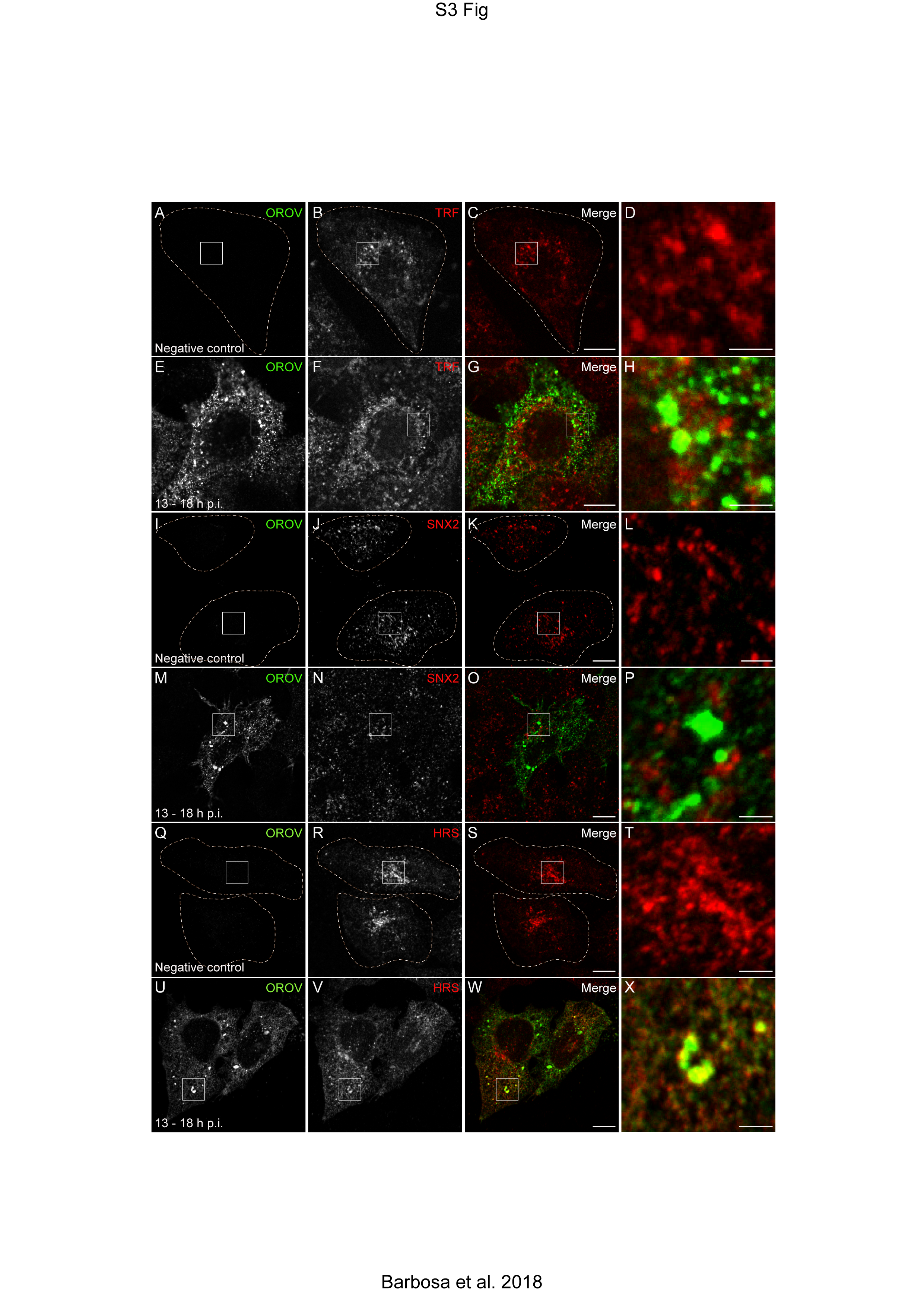

Supplement: S3 Fig — (A—H) Control or infected cells (MOI = 1) were incubated with transferrin-Alexa488 (TRF, red) for one hour and then fixed at the indicated time p.i.. Cells were stained with antibody to OROV proteins (green) and analyzed by immunofluorescence and confocal microscopy. (I—X) Control or OROV infected cells (MOI = 1), were fixed at indicated times p.i., double stained with antibodies to early endosome markers (HRS and SNX2, shown in red) and to OROV proteins (green) and analyzed by immunofluorescence and confocal microscopy. Cell outlines are indicated by dashed lines. Bars = 10 μm. (D, H, L, P, T and X) Insets representing the boxed areas of A-C, E-G, I-K, M-O, Q-S and U-W respectively. Bars = 2 μm. (TIF) [file ppat.1007047.s003.tif]

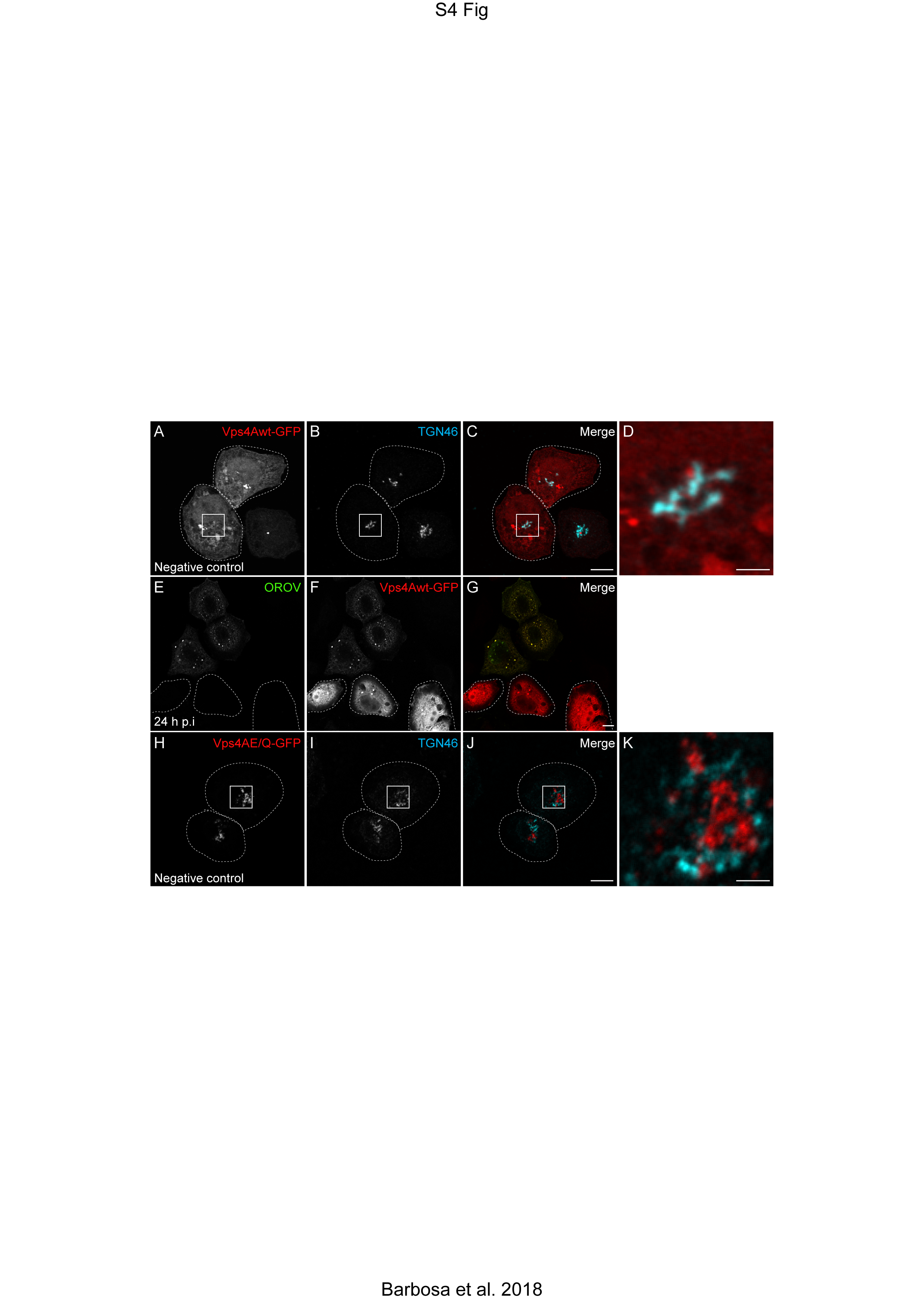

Supplement: S4 Fig — (A-D) Control HeLa cells expressing Vps4Awt-GFP (shown in red) were immunostained with an anti-TGN46 antibody (cyan). (E-G) HeLa cells were infected with OROV (MOI = 3) and then transfected with Vps4Awt-GFP plasmid. After 24 h of infection, cells were fixed, double stained with anti-OROV (shown in green to facilitate comparison with other Figures) and anti-TGN46 (Cyan) antibodies. (H-K) Control HeLa cells expressing Vps4E/Q-GFP (shown in red) were immunostained with an anti-TGN46 antibody (cyan). Cells were analyzed by immunofluorescence and confocal microscopy. Cell outlines are indicated by dashed lines. Bars = 10 μm. (D and K) Insets representing the boxed areas of A-C and H-J respectively. Bars = 2 μm. (TIF) [file ppat.1007047.s004.tif]

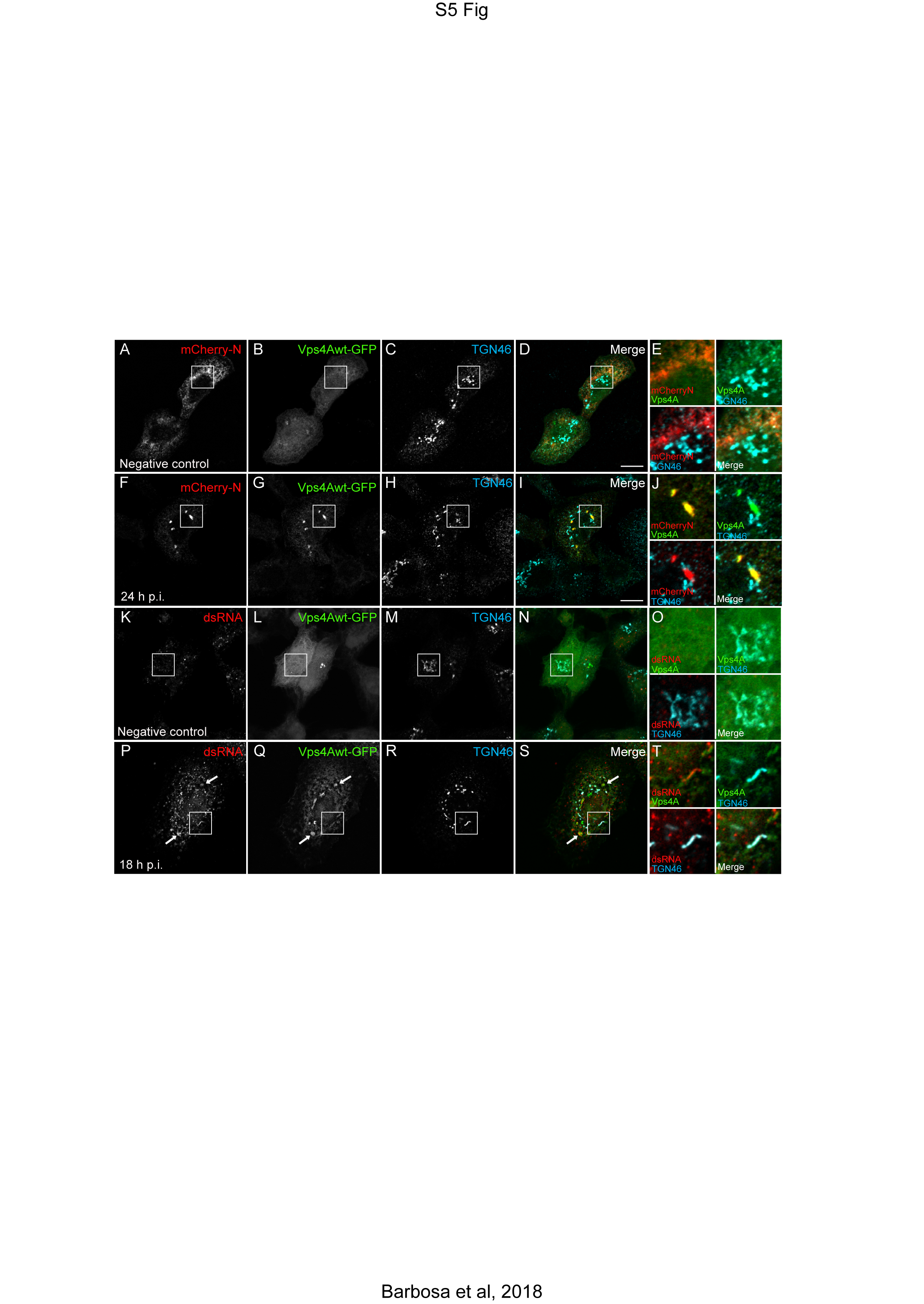

Supplement: S5 Fig — (A-E) Control HeLa cells expressing Vps4Awt-GFP and mCherry-N of OROV were immunostained with an anti-TGN46 antibody (cyan). (F-J) HeLa cells were infected with OROV (MOI = 3) and then transfected with referred plasmids. After 24 h p.i, cells were fixed and stained with anti-TGN46 (cyan) antibody. (K-O) Control HeLa cells expressing Vps4wt-GFP were immunostained with a J2 anti-dsRNA (red) and an anti-TGN46 (cyan) antibodies. (P-T) HeLa cells were infected with OROV (MOI = 3) and then transfected with Vps4Awt-GFP plasmid. After 18 h p.i, cells were fixed and costained with J2 anti-dsRNA (red) and anti-TGN46 (cyan) antibodies. Cells were analyzed by immunofluorescence and confocal microscopy. Cell outlines are indicated by dashed lines. Bars = 10 μm. (E, J, O and T) Insets representing the boxed areas of A-D, F-I, K-N and P-S respectively. Bars = 2 μm. (TIF) [file ppat.1007047.s005.tif]

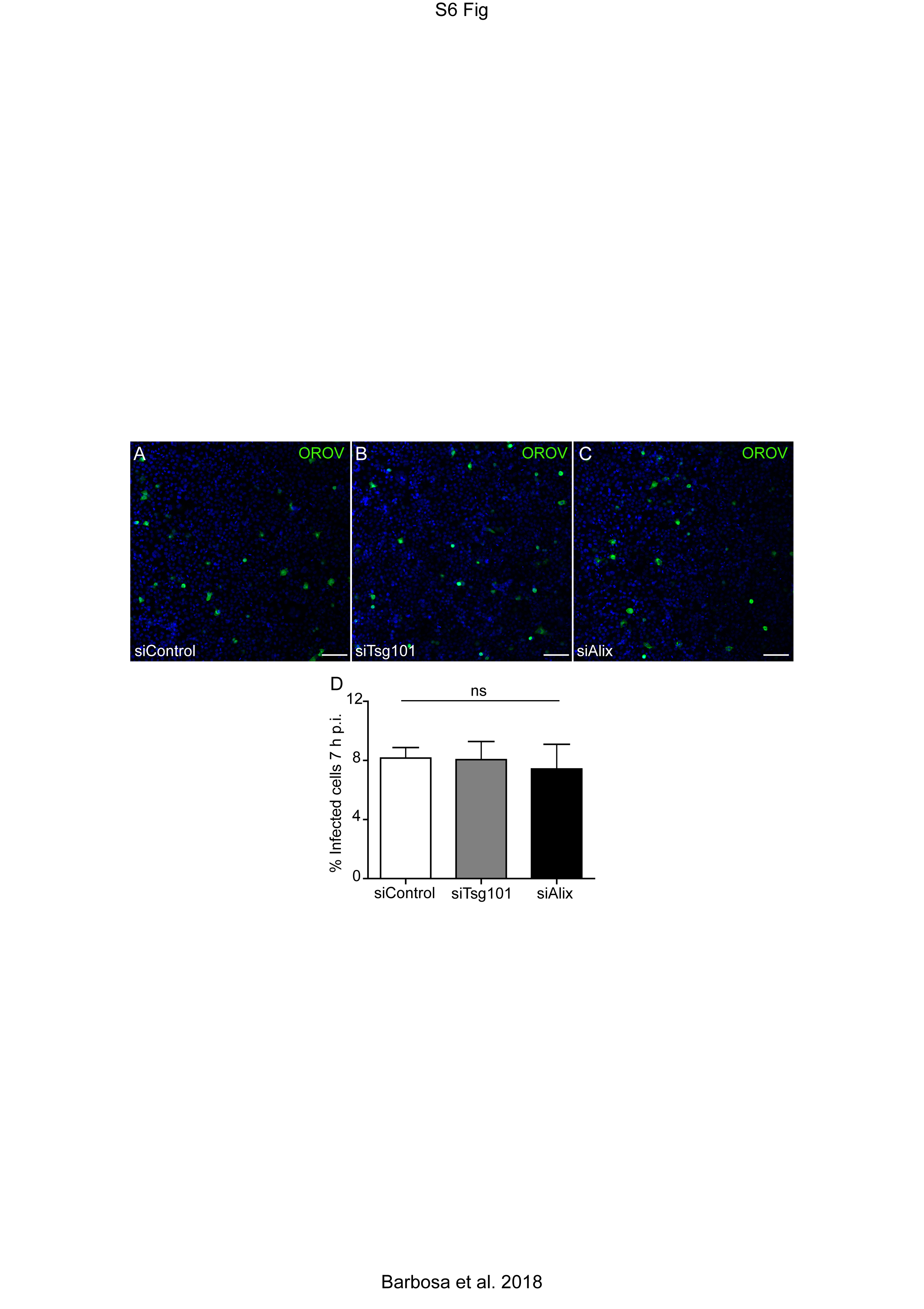

Supplement: S6 Fig — HeLa cells transfected with either control siRNA (A) or siRNA to Tsg101#1 (B) or Alix#1 (C) were infected with OROV (MOI = 3) for 7 h. Cells were immunostained with antibody to OROV proteins (green) and analyzed by conventional immunofluorescence microscope. Nuclei were stained with DAPI (blue). Bars = 100 μm. (D) The amount of infected cells (A–C) was calculated from the percentage of total cell count. ns–non significant (one-way ANOVA followed by Bonferroni post-test). (TIF) [file ppat.1007047.s006.tif]
