# Supplementary material for: ESCRT machinery components are required for Orthobunyavirus particle production in Golgi compartments
Source: PLoS Pathog. 2018 May 3;14(5):e1007047. doi: 10.1371/journal.ppat.1007047 (PMC5953487; doi:10.1371/journal.ppat.1007047)
Supplement: S1 Table — (DOCX) [file ppat.1007047.s007.docx]

|  |  | Mander’s | coefficient |  |
| --- | --- | --- | --- | --- |
| Proteins | Time p.i. | tM1 | tM2 | n |
| Calnexin – OROV (fig. S1) | 13 – 18 h | 0.37 ± 0.04 | 0.38 ± 0.03 | 5 |
| CD63 – OROV (fig. S2) | 13 – 18 h | 0.35 ± 0.09 | 0.25 ± 0.03 | 5 |
| Lamp-1 – OROV (fig S2) | 13 – 18 h | 0.1 ± 0.06 | 0.09 ± 0.04 | 5 |
| TRF – OROV (fig S3) | 13 – 18 h | 0.18 ± 0.06 | 0.26 ± 0.06 | 5 |
| HRS – OROV (fig. S3) | 13 – 18 h | 0.64 ± 0.27 | 0.60 ± 0.09 | 10 |
| SNX2 – OROV (fig. S3) | 13 – 18 h | 0.18 ± 0.10 | 0.40 ± 0.06 | 8 |
| Vps4AE/Q – OROV (fig. S4) | 0 h | not detected | not detected | 5 |
| Vps4AE/Q – TGN46 (fig. S4) | 0 h | 0.1 ± 0.03 | 0.075 ± 0.06 | 5 |
| Vps4AE/Q – OROV (fig. 4) | 24 h | 0.68 ± 0.33 | 0.60 ± 0.06 | 6 |
| Vps4AE/Q – TGN46 (fig. 4) | 24 h | 0.50 ± 0.12 | 0.48 ± 0.12 | 6 |
| TGN46 – OROV (fig. 4) | 24 h | 0.88 ± 0.10 | 0.73 ± 0.07 | 6 |
| Alix – OROV (fig. 7) | 0 h | not detected | not detected | 5 |
| Alix – TGN46 (fig.7) | 0 h | 0.05 ± 0.04 | 0.13 ± 0.10 | 5 |
| Alix - OROV (fig.7) | 24 h | 0.71 ± 0.21 | 0.64 ± 0.09 | 6 |
| Alix – TGN46 (fig.7) | 24 h | 0.47 ± 0.08 | 0.56 ± 0.10 | 6 |
| TGN46 – OROV (fig.7) | 24 h | 0.91 ± 0.10 | 0.82 ± 0.10 | 6 |
| N-mCherry – OROV (fig S5) | 24 h | 0.77 ± 0.16 | 0.51 ± 0.11 | 5 |
| dsRNA – Vps4wt (fig S5) | 18 h | 0.54 ± 0.06 | 0.5 ± 0.12 | 6 |

**SUPPLEMENTAL TABLE**

**S1 Table: Quantitative analysis of colocalization between OROV proteins and dsRNA, ER, TGN, or endosomal proteins^a^.**

^a^Quantification of colocalization was performed using the colocalization threshold plugin in the ImageJ software 1.49. Mander’s coefficient parameter evaluates the single-channel specific coefficients tM1 and tM2. tM1 coefficient is related to the overlap of the red channel (organelle marker staining) over the green channel (OROV staining) and tM2 represents the overlap of the green channel over the red channel. The table shows the mean ± SD of tM1 and tM2. Data of each pairwise comparison were obtained from Z-slices (0,3 µm intervals) of at least four cells from each experiment.
